# Degradation of Bio-Based and Biodegradable Plastic and Its Contribution to Soil Organic Carbon Stock

**DOI:** 10.3390/polym15030660

**Published:** 2023-01-28

**Authors:** Vusal Guliyev, Benjawan Tanunchai, Maria Udovenko, Oleg Menyailo, Bruno Glaser, Witoon Purahong, François Buscot, Evgenia Blagodatskaya

**Affiliations:** 1 Department of Soil Ecology, UFZ-Helmholtz Centre for Environmental Research, 06120 Halle (Saale), Germany; 2 Department of Biology, University of Leipzig, 04103 Leipzig, Germany; 3 Institute of Soil Science and Agro Chemistry, Azerbaijan National Academy of Science, AZ1073 Baku, Azerbaijan; 4 Bayreuth Center of Ecology and Environmental Research (BayCEER), University of Bayreuth, 95448 Bayreuth, Germany; 5 Joint FAO/IAEA Centre of Nuclear Techniques in Food and Agriculture, Soil and Water Management and Crop Nutrition Laboratory, 2444 Seibersdorf, Austria; 6 Department of Soil Biogeochemistry, Martin Luther University Halle-Wittenberg, 06120 Halle (Saale), Germany; 7 German Centre for Integrative Biodiversity Research (iDiv), Halle-Jena-Leipzig, 04103 Leipzig, Germany

**Keywords:** PBSA, soil respiration, bio-based and biodegradable plastic, isotopic fractionation, source partitioning, priming effect

## Abstract

Expanding the use of environmentally friendly materials to protect the environment is one of the key factors in maintaining a sustainable ecological balance. Poly(butylene succinate-co-adipate) (PBSA) is considered among the most promising bio-based and biodegradable plastics for the future with a high number of applications in soil and agriculture. Therefore, the decomposition process of PBSA and its consequences for the carbon stored in soil require careful monitoring. For the first time, the stable isotope technique was applied in the current study to partitioning plastic- and soil-originated C in the CO_2_ released during 80 days of PBSA decomposition in a Haplic Chernozem soil as dependent on nitrogen availability. The decomposition of the plastic was accompanied by the C loss from soil organic matter (SOM) through priming, which in turn was dependent on added N. Nitrogen facilitated PBSA decomposition and reduced the priming effect during the first 6 weeks of the experiment. During the 80 days of plastic decomposition, 30% and 49% of the released CO_2_ were PBSA-derived, while the amount of SOM-derived CO_2_ exceeded the corresponding controls by 100.2 and 132.3% in PBSA-amended soil without and with N fertilization, respectively. Finally, only 4.1% and 5.4% of the PBSA added into the soil was mineralized to CO_2_, in the treatments without and with N amendment, respectively.

## 1. Introduction

A large amount of plastic is applied to the packaging of commercial products such as food, pharmaceuticals, cosmetics and chemical products and in modern agriculture (e.g., mulching plastic films). The incineration of this traditional plastic waste releases a large amount of carbon dioxide, which contributes to global warming [1,2]. Plastic debris and the toxic gases produced as intermediate products of its degradation result in environmental pollution, thereby increasing the demand for consumers to use more natural and recyclable packaging materials [1,3]. Therefore, it is preferable and efficient to replace conventional plastics with a sustainable alternative, such as bioplastics, which are produced from renewable biomass sources. Expanding the use of environmentally friendly materials to protect the environment is one of the key factors in maintaining a sustainable ecological balance [4]. Currently, only one percent of total plastics production (368 million tons annually) is bioplastic, which by definition is bio-based, biodegradable, or features both properties [3]. Bio-based and biodegradable plastics (BBPs) are defined as consisting entirely or partly of biomaterial, which can be decomposed by microorganisms in the environment [5]. Plastics that can be completely decomposed into carbon dioxide and water within a certain period of time by naturally occurring microorganisms are considered ideal biodegradable plastics [6]. In comparison to conventional plastics, bio-based and biodegradable plastics offer multiple benefits, including saving fossil fuels, reducing greenhouse gas emissions, avoiding microplastic residues in the environment and improving carbon turnover [7,8]. The popularity of bio-based and biodegradable plastics such as plant-derived poly(butylene succinate) PBS, poly(butylene succinate-co-adipate) PBSA and poly(butylene adipate-co-terephthalate) PBAT has grown due to their potential to mitigate the problems arising from the use of synthetic plastics [9,10,11]. The use of plants such as maize and sugarcane in plastic polymerization enables the monitoring of plastic decomposition by measuring the ^13^C signature [1,12].

Increasing food consumption, which requires more efficient and sustainable agriculture, makes plastic mulching more essential to increase crop yields and improve crop quality [13,14]. At the end of use, the plastic mulch cannot be completely removed from the soil without leaving any residue [15,16]. Thus, micro- and nanoplastic fragments are disposed of in an environment where they can remain for a long time and negatively affect aquatic and terrestrial ecosystems [17]. Plastic fragments can impair digestive functions when consumed by aquatic and terrestrial animals [18]. The impact of microplastics on terrestrial systems depends on the type of microplastic and the plant–soil system involved [11,19]. Further phases of any soil modification would be detrimental to health since soils are environmental systems that can carry toxins to plants and the human food chain. Microplastics can alter soil structure and bulk density, obstructing root penetration, water flow and other processes. Exposed to sunlight and active microorganisms, fragmented plastics release toxic additives into the soil that can affect plant growth and microbial activity [20,21].

The degradation rate of BBPs depends on the shape, size and type of plastic and the soil ecosystem [3,22]. After mechanical degradation, the fragmented plastics are attacked by microorganisms, which release enzymes to catalyze polymer transformation into oligomers or monomers [7]. In this way, small molecules penetrate the microbial cell where they are metabolized and subsequently released as end products such as CO_2_, CH_4_, H_2_O and N_2_. Given the complexity of the chemical composition of plastics, their decomposition requires the interactive activity of the appropriate microbial consortia rather than individual taxa. Microalgae-based consortia, for example, provide effective and environmentally friendly solutions for the biological breakdown of plastics in aquatic systems [23]. The microalgae colonization of the plastic surface and their ability to metabolize plastic in the aquatic environment make their use environmentally safe and promising for addressing the issues of plastic waste [24]. In comparison to traditional plastics, BBPs are more effective at reducing the greenhouse effect of plastics [25,26,27] and microplastic residues in soils [28]. 

The fresh input of available C switches soil microorganisms from dormant to active stages [29]. The stimulated microbial activity can cause a change in the SOM decomposition rate [30]. This alteration, known as the priming effect (PE), either decreases (negative PE) or increases (positive PE) the decomposition rate of SOM, which is explained by several mechanisms such as “preferential utilization”, “microbial N-mining”, “stoichiometric decomposition”, etc. [31,32]. The duration and intensity of PE and the contribution of microorganisms to SOM decomposition are controlled by N availability [33,34]. Since the BBPs are C-rich and N-depleted, the decomposition of stoichiometrically imbalanced plastics with a large C: N ratio in soil might increase the SOM degradation rate [35]. Therefore, the fertilization of soil contaminated by BBPs can be potentially used to decrease the PE, i.e., resulting in a decrease in the SOM decomposition rate and increase in BBP decomposition [36]. Therefore, it is necessary to consider PE and the transformation of the SOM in the studies on BBP decomposition in soil.

Among several techniques to track the decomposition of plastics [1,37], stable isotope analysis is very promising to distinguish the origin of the intermediate products of plastic decomposition in the case of difference in isotopic signature between plastics and SOM [38,39,40]. The use of naturally occurring ^13^C tracers in soil studies is based on the different photosynthetic pathways between C3 and C4 plants [41]. The δ^13^C values of C4 plants, such as maize or sugarcane, range from −12‰ to −10‰ [42]. C4 plant-derived products demonstrate higher ^13^C content than C3 plants. Thus, an application of C4-bio-based plastics to a C3 soil enables the identification of the origin of the end products. As plastic decomposition is monitored indirectly by the CO_2_ emission, the possible shift in the ^13^C signature of the SOM and its mineralization products due to: (i) ^13^C isotopic fractionation, i.e., the difference between heavy and light coexisting isotopes of an element; or (ii) ^13^C isotopic discrimination (see below) needs to be considered in the calculations [40]. Such a shift is caused either by: (i) the preferential use of substances with light ^12^C versus heavy ^13^C [43]; or (ii) by the preferential utilization of heavy ^13^C-substrate of high availability (sugars, cellulose) versus the light ^12^C of low availability (lignin, lipids) [44]. The discrimination between SOM-C and SOM-respired CO_2_ can be ^13^C-enriched, near-zero difference and ^13^C-depleted [45,46,47,48]. Since the N is the driver for the microbial utilization, its availability might strongly affect the carbon isotope fractionation in microbial processes [49,50]. 

Our study attempted to distinguish plastics and soil C sources in the course of the decomposition of bio-based and bio-degradable plastics using stable isotope techniques. For this purpose, we used the isotope partitioning approach to differentiate the plastic-derived CO_2_ from the soil-derived CO_2_. Our aim was to determine: (i) the degradation rate of BBPs by the contribution of plastic-derived C to the CO_2_ emission; (ii) the effect of N fertilization on the plastic degradation rate: and (iii) the priming effect of soil organic matter induced by plastic addition, using δ^13^CO_2_ source partitioning. We hypothesize that: (i) adding N (as a limiting nutrient in the BBPs) significantly increases plastic-derived CO_2_ emission as the biodegradation of BBPs increases; (ii) BBPs are a potential C source that can cause PE.

## 2. Materials and Methods

### 2.1. Material

We tested the renewable-based PBSA (poly(butylene succinate-co-adipate)) as one of the most promising and new biopolymers. The PBSA is the combination of succinic acid and adipate acid with 1-4-butanediol. The PBSA plastic films were purchased from PTT MCC Biochem Company Limited (Thailand) with the BioPBS FD92 trade name [8,11]. The 50 μm thick, double-layered PBSA film contains 55% C and consists of 35% bio-based carbon. Before being applied to soil, the PBSA was sterilized with 70% ethanol and cut into small pieces (2–5 mm × 2–5 mm). The δ^13^C of PBSA was determined by elemental analysis coupled to an isotope ratio mass spectrometer (Delta Plus XL IRMS, Thermo Finnigan MAT, Bremen, Germany).

### 2.2. Design of Experiment

The laboratory experiment was conducted on soil sampled from a conventional farming plot of the Global Change Experimental Facility (GCEF), Bad Lauchstädt, Central Germany (51°22′60 N, 11°50′60 E, 118 m a.s.l.). The soil was classified as a Haplic Chernozem with water-holding capacity = 35%, total organic C = 2%, C: N ratio = 10, pH = 7. 5 and silt-loam soil texture with a 70% silt, 20% clay and 10% sand combination [51,52]. Considering the δ^13^C of PBSA used, the conventional farming plot was chosen with most distinct difference in δ^13^C between SOM and PBSA. The plot is under a typical regional crop rotation consisting of winter rape, winter wheat and winter barley [53]. The soil was sampled from winter barley, homogenized passing through a 2 mm sieve and divided into the following treatments: (1) control soil without PBSA (S); (2) control soil without PBSA and with (NH_4_)_2_SO_4_ addition (SN); (3) PBSA–soil—soil with PBSA addition (PS); (4) PBSA–soil–N—soil with PBSA and (NH_4_)_2_SO_4_ addition (PSN). The PBSA treatment contained 28.5 g of soil (equivalent to 23.5 g dry soil) and 1.5 g (6% PBSA, *w*/*w*) of plastic material [11,53,54]. The five replicates of each treatment were put into a glass container and placed into plastic 250 mL jars in the respirometer for CO_2_ emission measurement. In the PSN and control SN treatments, 0.0825 g N was added in soil as 2.1 mL of 1.42 M (NH_4_)_2_SO_4_ to maintain C: N ratio (10:1) and simulate fertilization in agricultural system. For PS and control treatments (without N), 2.1 mL sterile Milli-Q water was added to equalize water content as in the N added treatments (17.5%, accounting for 50% of the water-holding capacity). All treatments were incubated at a constant soil moisture and air temperature (22 °C) during 80 days of the experiment.

### 2.3. Soil Respiration

Soil respiration was determined using an automated respirometer Respicond V (Nordgren Innovations, Sweden) during 80 days at 22 °C. The system enabled a continuous measurement of CO_2_ trapped by 0.6 M KOH by a decrease in electrical conductivity of KOH solution [55]. During the experiment, the KOH with the trapped CO_2_ was collected to measure δ^13^C in dynamics. Thereafter, a new KOH was added to the system at each sampling time.

### 2.4. Source-Partitioning of CO_2_ Emitted from Soil and from PBSA

Samples for determination of δ^13^C signature in CO_2_ were obtained by C precipitation [40]. At first, the CO_2_ trapped as K_2_CO_3_ in 10 mL KOH was precipitated with 15 mL of 0.5 M BaCl_2_ aqueous solution. Then, the KOH solutions containing the BaCO_3_ precipitate were centrifuged three times at 3000× *g* for 5 min and washed in between with deionized and degassed water to remove an excess of KOH and to reach a pH of 7. After washing, the supernatant water was removed from the vials and the BaCO_3_ was dried at 105 °C for 24 h. The solid samples of BaCO_3_ were analyzed for δ^13^C values by elemental analysis coupled to an isotope ratio mass spectrometer (Delta Plus XL IRMS, Thermo Finnigan MAT, Bremen, Germany).

**Figure 1:**
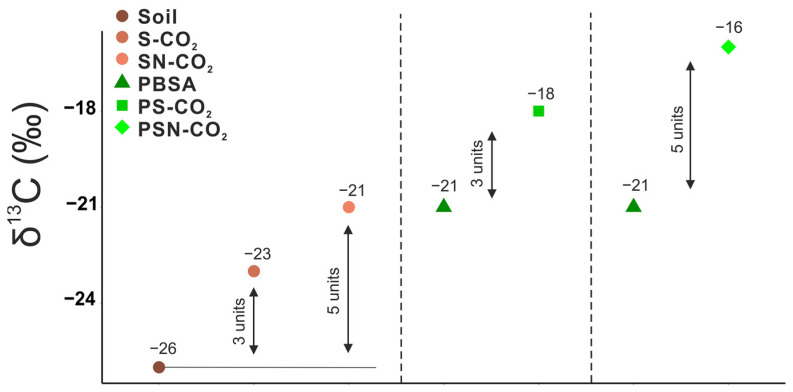
Isotope signature of ^13^C sources: Soil—δ^13^C in soil, S-CO_2_—δ^13^C in CO_2_ respired from soil without N addition, SN-CO_2_—δ^13^C in CO_2_ respired from soil with N addition, PBSA—δ^13^C of PBSA, PS-CO_2_—an estimated shift in δ^13^C of PBSA in CO_2_ respired from soil in the PS treatment; PSN-CO_2_—an estimated shift in δ^13^C of PBSA in CO_2_ respired from soil in PSN treatment.

Isotope fractionation between soil-C and CO_2_-C was estimated as difference between the δ^13^C value of soil (−26‰) and the corresponding δ^13^C value of CO_2_-C released from this control soil (−23‰). ^13^C enrichment in respired soil CO_2_ is a common phenomenon and can be explained by preferential use of ^13^C-enriched SOM compounds such as sugars [41]. The N effect on ^13^C fractionation was considered as a difference between δ^13^C of CO_2_-C from control soil with and without added N (Figure 1). The isotope fractionation accounted for 3‰ (S) and 5‰ (SN) as compared to the δ^13^C of corresponding SOM. Thus, N addition further enriched δ^13^C of CO_2_-C by 2‰ compared to SOM mineralization without additional N. The further δ^13^C source partitioning was based on the assumption of similar isotope fractionation of CO_2_-C originated from both soil and plastics [42,56]. 

The δ^13^C notation expresses carbon isotope ratios of sample and standard [38,57]:

(1) $$\delta^{13}C\left(‰\right)=(((\delta^{13}C/\delta^{12}C_{\mathrm{sample}})/(\delta^{13}C/\delta^{12}C_{\mathrm{standard}}))-1)\times 1000$$

where δ^13^C is the parts per thousand difference between the ^13^C content of the sample and the standard. The δ^13^C values are expressed relative to the reference standard Vienna-PeeDee Belemnite (VPDB), being equal to 0.00112372.

The fraction of plastics-derived C in total C in CO_2_ was estimated according to Amelung et al. [38]:

(2) $$F_{\mathrm{PBSA}}=(\delta^{13}C_{t}-\delta^{13}C_{\mathrm{cs}})/(\delta^{13}C_{\mathrm{PBSA}}-\delta^{13}C_{\mathrm{cs}})$$

where δ^13^C_t_ is the δ^13^C value of the CO_2_-C released from the PBSA-amended soil; δ^13^C_cs_ is the δ^13^C value of coinciding CO_2_-C pool, which is referred to control soil; δ^13^C_PBSA_ is the δ^13^C value of PBSA.

Before the CO_2_ source partitioning, we corrected the δ^13^C_cs_ by the CO_2_ possibly entrapped from the air:

(3) $$\delta^{13}C_{\mathrm{cs}}=\left(\left(C_{\mathrm{cs}+a}{\times \delta }^{13}C_{\mathrm{cs}+a}\right)-\left(C_{a}{\times \delta }^{13}C_{a}\right)\right)/\left(C_{\mathrm{cs}+a}-C_{a}\right)\;$$

where the C_cs_ and C_a_ are the amount of C derived from soil-respired CO_2_ and atmospheric CO_2_, respectively; the C_cs+a_ is value of the mixture of C derived from soil-respired CO_2_ and from atmospheric CO_2_ in the incubation jar; δ^13^C_a_ is the δ^13^C value of the air CO_2_; the δ^13^C_cs+a_ is the δ^13^C value of the mixture of CO_2_ originated from soil and from air in the incubation jar. 

The amount of plastics mineralized to CO_2_ was calculated according to the following equation:

(4) $$C_{\mathrm{PBSA}-\mathrm{derived}}=C_{t}{\times F}_{\mathrm{PBSA}}$$

where C_t_ is the total amount of CO_2_-C released from PBSA-amended soil.

To estimate priming effect (PE), we used the mass balance equation [40,57]:

(5) $$\delta^{13}{C\times \mathrm{CO}}_{2}\text{-}C_{\mathrm{total}}=\delta^{13}{C\times \mathrm{CO}}_{2}\text{-}C_{\mathrm{PBSA}})+\left(\delta^{13}{C\times \mathrm{CO}}_{2}\text{-}C_{\mathrm{soil}}\right)+(\delta^{13}{C\times \mathrm{CO}}_{2}\text{-}C_{\mathrm{PE}})$$

(6) $$\delta^{13}{C\times \mathrm{CO}}_{2}\text{-}C_{\mathrm{PE}}=(\delta^{13}{C\times \mathrm{CO}}_{2}\text{-}C_{\mathrm{total}})-\left(\delta^{13}{C\times \mathrm{CO}}_{2}\text{-}C_{\mathrm{PBSA}}\right)-(\delta^{13}{C\times \mathrm{CO}}_{2}\text{-}C_{\mathrm{soil}})$$

where the CO_2_-C is an amount of C in the corresponding pool, i.e., “PBSA” indicates C derived from PBSA, “soil”—C derived from soil, “PE” is the priming effect and “total” is the sum of all pools. 

### 2.5. Statistical Analysis

The open-source R software version 3.6.1 was used to perform all statistical analysis. The effects of soil treatments on CO_2_ emission and fractions were analyzed using analysis of variance (ANOVA), incorporating the Shapiro–Wilk test for normality and Levene’s test to assess the equality of group variances. If variance homogeneity and normality were given, an ANOVA was conducted by following the Tukey post hoc test to compare the means with the *p* < 0.05 level of significance. Otherwise, the Kruskal–Wallis rank sum test served as a non-parametric alternative by following Dunn’s test as a post hoc test to compare the treatments for significant differences of multiple comparisons. Barplots were used to illustrate the data to display the distribution and changes in the variables. Since the values were used from different devices, the propagation of error was calculated to perform variation between replicates.

## 3. Results

### 3.1. CO_2_-C Respired

**Figure 2:**
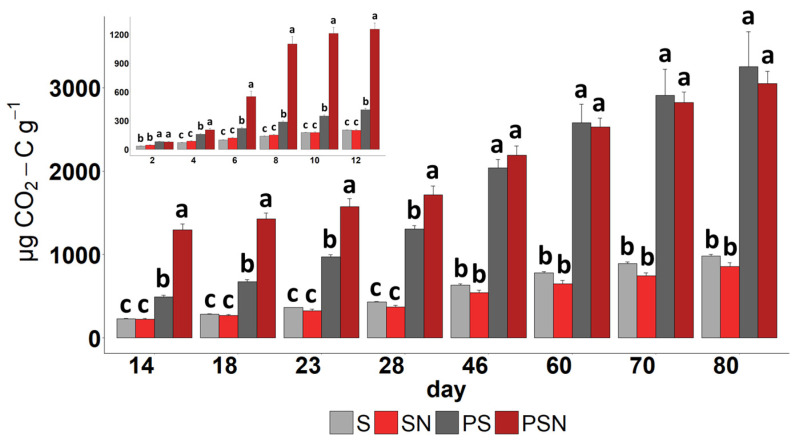
Cumulative amount of respired carbon during experiment. Inset figure demonstrates the first 12 days of experiment. S indicates control soil without N addition, SN—control soil with N addition, PS—PBSA-amended soil without N addition and PSN—PBSA-amended soil with N addition. Inserted figure’s units are corresponded to main figure. Different letters on the bars demonstrate significant differences between treatments within the sampling date.

The N addition to the control soil reduced the CO_2_ emission by 13% at the end of the experiment. From the beginning of the experiment, PBSA-amended soils respired greater amounts of CO_2_ as compared with the corresponding non-amended controls (Figure 2; Table S1). The respiration of the PBSA-amended soil without N gradually increased. After a week of the experiment, the N raised the CO_2_ efflux by 3.5 times in PSN as compared to PS treatment (Figure 2 insert). Larger amounts of CO_2_ were respired from the PSN versus the PS treatment until 28 days and thereafter the N effect was insignificant. In the first two weeks of PBSA decomposition, the CO_2_ released from the PS and PSN treatments exceeded that from control by the factors of 2 and 6, respectively. 

### 3.2. ^13^C Signature of Respired CO_2_-C

**Figure 3:**
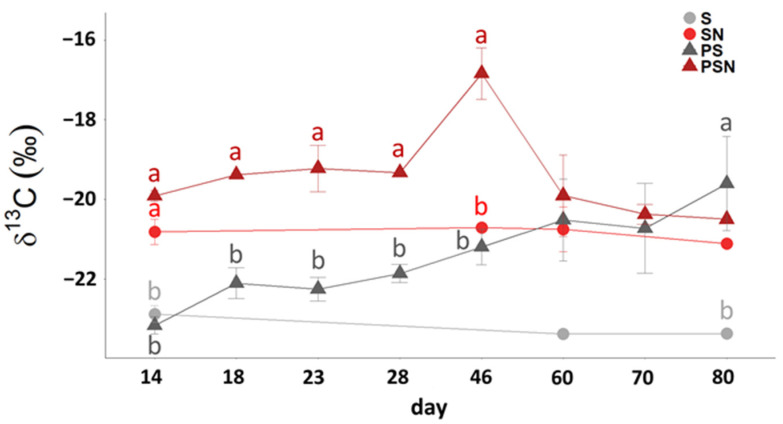
Dynamics of δ^13^CO_2_-C during experiment: S indicates control soil without N addition, SN—control soil with N addition, PS—PBSA-amended soil without N addition and PSN—PBSA-amended soil with N addition. The letters indicate significant differences between treatments at the corresponding time point.

The δ^13^CO_2_-C values of the control soils (S and SN) were relatively constant during the experiment (Figure 3). A significant N fertilization effect resulting in a 2‰ greater ^13^C enrichment of the CO_2_ respired from the SN versus S treatment was stable throughout the experiment (Figure 3). The ^13^C signature in the PS treatment gradually shifted towards the δ^13^C signature of the PBSA during the experiment (Figure 3). The δ^13^CO_2_-C in the PSN treatment was 3‰ more ^13^C enriched as compared to soil without N addition and it reached its highest level at 6 weeks of the incubation. After 6 weeks, the δ^13^C signature in the PSN treatment returned to the original level and decreased slightly for the rest of the experiment (Figure 3).

### 3.3. Source Partitioning of the Respired CO_2_-C

**Figure 4:**
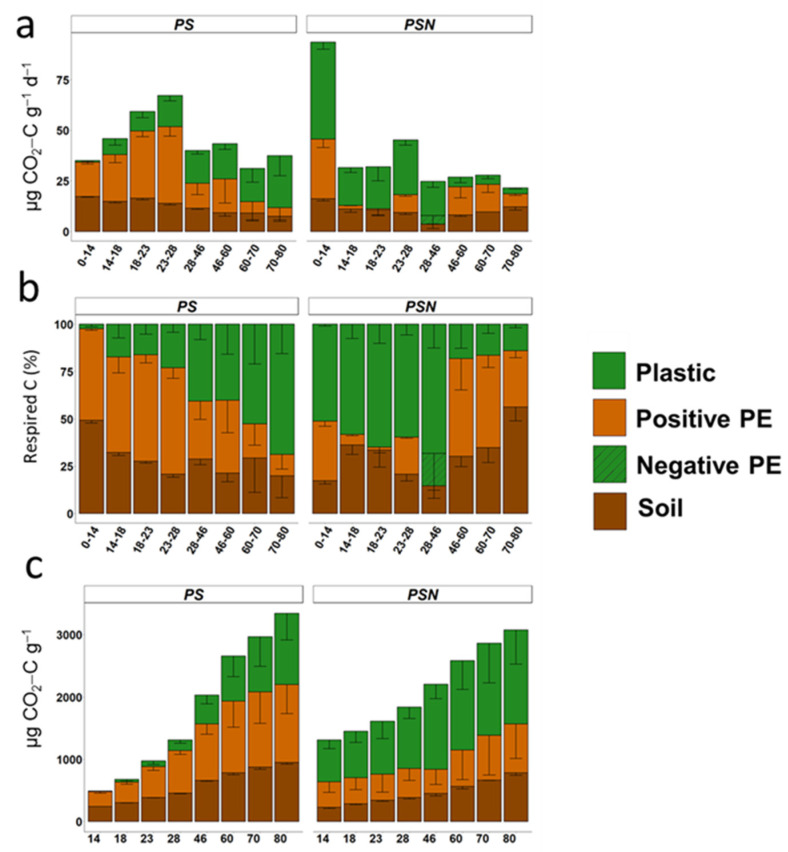
Respired CO_2_-C during 80 days of plastic decomposition: (**a**) absolute amount respired from previous sampling, i.e., between two subsequent samplings; (**b**) based on percentage of fractions at each time point; (**c**) cumulative amount of total CO_2_-C respired at each time point. Plastic—plastic-derived C fraction in CO_2_; Positive PE—positive priming effect fraction in CO_2_; Negative PE—negative priming effect fraction in CO_2_; Soil—CO_2_, released from corresponding control treatment without plastic.

Despite the fact that PBSA was degraded both with and without N addition, the rate of decomposition was not constant. Without N, PBSA was decomposed relatively slowly during the first month of the experiment, whereas N addition accelerated the decomposition rate in the first 4 weeks (Figure 2). The decomposition rate of PBSA was 57 times higher in the PSN versus the PS treatment after 14 days of incubation (Figure 4a; Table S2). The 28–46-day period of the experiment was a tipping point, after which the decomposition rate of PBSA in N-amended soil decreased dramatically. In terms of absolute values, PBSA decomposition gradually increased during the experiment when no N was added (Figure 4a). The decomposition of plastic in the PSN treatment was most intensive in the first 2 weeks of the experiment and strongly decreased after 60 days of incubation (Figure 4). 

In terms of relative values, the proportion of CO_2_ factions originated from soil and PBSA were strongly affected by the treatment and incubation time (Figure 4b; Table S3). In the PS treatment, the PBSA fraction in the respired CO_2_ generally increased over time (Figure 4b). The N amendment increased the PBSA fraction in CO_2_ by almost 21 times (Figure 4b). The two decomposition phases of PBSA, intensive (up to 6 weeks) and relatively slow, were distinguished in the PSN treatment. The largest contribution of PBSA-originated C to the CO_2_ respired was observed between 4 and 6 weeks of PBSA decomposition when N was added. After 6 weeks, however, the PBSA fraction in the CO_2_ emission decreased 4.7 times compared to the previous period. 

**Figure 5:**
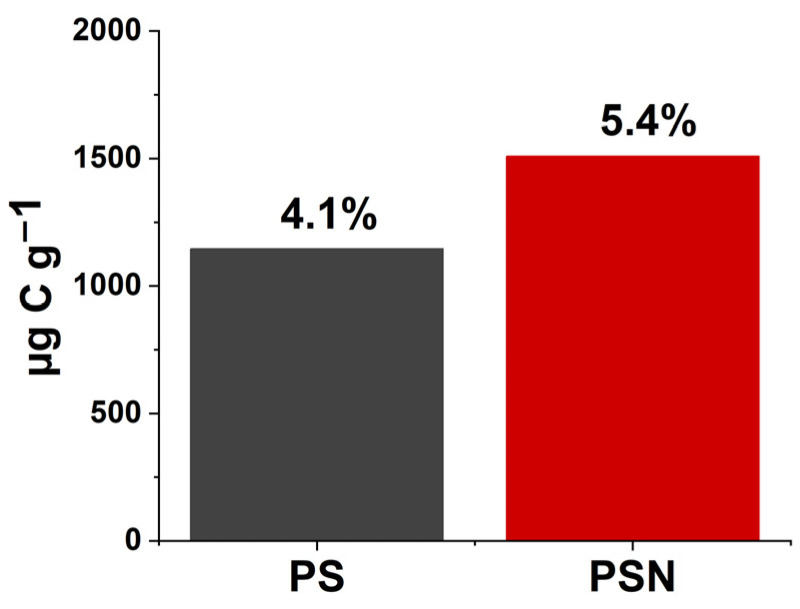
Cumulative amount of plastic-originated C mineralized to CO_2_. The values above the bars correspond to the percent of respired C in relation to the added C with PBSA.

Despite insignificant differences in the total respired CO_2_-C between PSN and PS (Figure 2), the contribution of PBSA was larger by 22% in the PSN versus the PS treatment (Figure 4c). At the end of experiment, only about 4.1% and 5.4% of the PBSA-C added in the PS and PSN treatments, respectively, were mineralized to CO_2_ (Figure 5).

### 3.4. Priming Effect

Essentially, a large fraction of primed soil C was detected in both treatments. The largest fraction of primed C was detected after 60 days in the PSN while this was not a case in the PS treatment (Figure 4b). The highest intensity of PE in the PS and PSN treatments was detected at 3 and 2 weeks after PBSA application to the soil, respectively. After reaching the highest SOM decomposition rate by PE in the PS treatment, it decreased towards the end of the incubation time. The intensity of the priming effect in the PS treatment decreased by 5 times between 3 and 11 weeks of plastic decomposition (Figure 4a). In contrast, the fraction of PE in the PSN treatment increased during the slow (after 46 days) as compared to the fast decomposition phase of plastic (Figure 4b), whereas the highest SOM decomposition rate was observed by primed C after 2 weeks. At 46 days of incubation, the decomposition of PBSA in the PSN treatment resulted in negative PE, which indicated 17% retarded SOM decomposition. Due to the consistently high fraction of PE in the PS treatment in the course of PBSA decomposition, the cumulative PE during 80 days of PBSA decomposition was 25% greater in the PS vs. the PSN treatment (Figure 4c). Remarkably, the C losses from soil by PE were comparable (PSN) or even exceeded by the factor of 1.3 (PS) the losses by basal respiration of the control soils.

## 4. Discussion

The 3‰ and 5‰ ^13^C-enriched CO_2_ from control soil without and with N indicated isotope fractionation (Figure 1). Isotope fractionation between soil and soil-respired CO_2_ is the result of two fractionation steps: the first isotope discrimination occurs during the metabolic processes, which leads to the preferential use of light ^12^C for the synthesized compounds and results in the ^13^C enrichment of the residual compounds, and the second fractionation is related to specific compound utilization by microorganisms [58,59,60]. The ^13^CO_2_-C enrichment during the utilization of complex soil organic matter can be explained by the preferential utilization of specific compounds strongly varying in ^13^C values [61,62]. The selective utilization of ^13^C-rich organic compounds such as sugars can cause an enrichment of ^13^C in soil-respired CO_2_ during heterotrophic respiration [42,45]. As living plants were removed from the soil, we assume that only microbial (heterotrophic) respiration was the source of soil-originated CO_2_. The quality of carbon that cycled between organic debris and biomass in the course of the microbial re-utilization of C differs from the originally assimilated carbon [63,64]. We demonstrated that N availability affected the microbial metabolic pathways of isotope discrimination and fractionation. At a low C: N ratio, soil microorganisms preferentially utilize easily decomposable SOM with ^13^C-enriched organic compounds such as sugars, starch, cellulose, proteins, or organic acids. Therefore, the difference between soil-C and CO_2_-C was much lower in non-amended versus N-amended soil (3‰ versus 5‰). 

The pattern of shifts in ^13^C values towards PBSA in PBSA-added soils and without PBSA-added soils are confirmed by other studies [37,65]. However, compared to other BBP investigations, the ^13^C values of PBSA-added soil shifted more quickly toward PBSA. In our study, the shifts in the PBSA-added soil with and without the addition of N were 3.7‰ and 2.0‰ after 6 weeks, respectively. The differences in the decomposition of PBSA between N addition in both scenarios (with and without N amendment) indicated a nutrient limitation in the soil. The nitrogen amendment stimulated the degradation of easily decomposable organic material, which contained ^13^C-enriched C compounds. The differences between the ^13^C signature of the PBSA-amended soil without and with N fertilization indicated that N availability changes the pathway of the decomposition of PBSA. The slowly increasing PBSA degradation when no N was added can be explained by the lack of N. Since PBSA does not contain nitrogen, the PBSA addition to soil increases the C:N ratio and the N deficiency in the PBSA-soil system. Microorganisms are able to colonize the plastic films, but because of the N requirement they are not efficient in PBSA degradation [8]. As the microorganisms that colonized the polymer surfaces [12] require N to grow [66], we assume that the microorganisms in the PS treatment without the N addition invested an energy from the carbon source to mine for N from SOM. Therefore, we assume that in the case of pure PBSA addition, the decomposition of PBSA was slowly fueled by N obtained through priming. Priming in such a case could serve as an indication of microbial growth. An alternative N source could be gained through N-fixing bacteria, which contributed to N acquisition in the PS treatment [8]. In the N-amended PBSA treatment, the PBSA decomposition rate was faster from the beginning of the experiment until the N requirements were met. However, a reduced CO_2_ emission rate and increased PE after 46 days in PSN treatment could be an indication of N limitation in this treatment in the last 5 weeks of plastic decomposition [8,11]. 

The input of PBSA served as a C source in both treatments [67,68] and altered the CO_2_ release from the soil causing a priming effect. Indeed, the PE, in other words the stimulation of SOM mineralization, was stronger in the N-poor PS treatment during the first 4 weeks as compared to the remainder of the experiment. Thus, the N requirements of microorganisms involved in PBSA degradation were covered by intensive SOM mineralization, which proves the “N-mining” mechanisms of PE. The PE during the 2 week period of the experiment in the PSN treatment can be explained by “stoichiometric decomposition” mechanisms, which are related to the increasing biomass and enzymatic activity at high C and N availability. Our assumption in the case of N fertilization is that an input of C and N matches microbial demands at the early stage of PBSA decomposition. In this case, microbial decomposers release extracellular enzymes, which are able to break down SOM along with PBSA. Thus, microbial activity increased the losses of the soil-originated CO_2_–C. The strong decrease in PE by a factor of 16 after two weeks and even the negative PE observed during days 28–46 of the experiment (Figure 4b) compared with the first two weeks of the PSN treatment indicated the microbial switch from SOM decomposition to PBSA utilization, which can be explained by shifting from “stoichiometric decomposition” to “preferential utilization” mechanisms. At the later decomposition stage, however, when added N was utilized, microorganisms growing on PBSA faced the lack of N and possibly of other nutrients. We assume, therefore, that the “tipping point” of 46 days can be explained by shifting from “preferential utilization” to a “N-mining” mechanism due to the depletion of the added N. 

The contrasting dynamics of PE in the treatments with and without N could also indicate the successional changes in the community of PBSA decomposers as well as corresponding changes in dominating microbial taxa. This was supported by the observed assembly of archaeal (Nitrososphaeraceae), bacterial (e.g., N fixing bacteria *Bradyrhizobium* spp.) and fungal (Sordariomycetes, Eurotiomycetes, Leotiomycetes) taxa dominating plastic decomposition under the lack of N [53]. In contrast, N-fixing bacteria disappeared from the N-amended PBSA decomposition and the community domination shifted towards nitrophilic microorganisms such as ammonia-oxidizing archaea (Nitrocosmicus and Nitrososphaeraceae) and denitrifying bacteria (*Achromobacter insolitus*, *Achromobacter denitrificans*) [8].

In total, 4.1% and 5.4% of the added PBSA was respired as CO_2_ without and with N-amended soils, respectively, during 80 days, which is consistent with other PBSA decomposition studies [11,27]. However, the mineralization of PBSA to CO_2_ is much lower as compared to the mineralization of natural substrates in soil, whereas PBSA-induced primed C is near the highest border of natural source-induced primed C (Table 1). The degradation of BBPs has been demonstrated by the weight loss approach with other studies, whereas the fate of PBSA is unknown: whether it degraded into micro- and/or nano-plastics or became part of the biomass and mineralized into CO_2_. In our study, we used the “source partitioning of the CO_2_-C respired” approach to trace the decomposition of BBPs, which is more relevant to identifying the fate of plastics. Assuming the carbon use efficiency ≈ 50–60% [69], we consider that roughly 9–10% of the BBPs were degraded during 80 days. That means about 75 mg g^−1^ of added plastic was either incorporated in the microbial biomass or transformed into secondary metabolites and micro-size particles, while 1350 mg g^−1^ of plastic remained unaffected. N accelerated the degradation of plastic at least during the period of 80 days, as shown by the 31.6% greater PBSA decomposition in the PSN as compared to the PS treatment, but this tendency retarded with time due to nutrient limitation.

## 5. Conclusions

We demonstrated the possibility of estimating the contribution of PBSA-derived C in CO_2_ by a stable isotope partitioning approach. The PBSA increased both the CO_2_ release and SOM mineralization, which may have been caused by increased microbial activity. In both cases—with and without N fertilization—the PBSA decomposition raised the overall CO_2_ output by a factor of around 2.5. However, the decomposition rate of PBSA depended on N availability. The N fertilization increased PBSA mineralization to CO_2_ by 31.6% and decreased the priming effect by 36% compared with the non-N treatment. Therefore, N fertilization coupled with optimal watering (to ensure appropriate redox conditions) could be recommended as an environmentally friendly strategy to facilitate plastic decomposition and reduce soil C and N_2_O losses under field conditions. Since the biodegradability of plastics is dependent on their quality, soil type and environmental conditions, the strength and duration of PE needs to be investigated in the future with a combination of various soils and plastics to reveal the mechanisms of C sequestration in soil.

## Figures and Tables

**Table 1:** Mineralization of different substrates in the soil environment and their effect on the soil organic matter (SOM) decomposition.

Labeled Substrate	Soil Type/Texture	Fraction of Substrate in Soil (%)	Mineralization to CO_2_ (% of Added)	Duration (Day)	Primed C to CO_2_ (% of Control)	Reference
^13^C Leaf (wheat)	Luvisol	0.5–1	46–54	120	31–42	[70]
^13^C Leaf (maize)	Alluvial, sand silt	0.6	13–19	32	44–67	[71]
^13^C Stem (wheat)	Luvisol	0.5–1	38–49	120	66–68	[70]
^13^C Root (wheat)	Luvisol	0.5–1	29	120	65–89	[70]
^13^C Root (tree)	Mollisol	0.6	13–19	85	5–31	[72]
^13^C Leaf litter	Ultisols (broad-leaved forest)	5	10–12	42	(−7)–9	[73]
^13^C Leaf litter	Ultisols (coniferous forest)	5	10–12	42	6–25	[73]
^13^C Leaf litter	Clay loam	5	24	125	(−7)–25	[74]
^14^C Glucose	Gleyic Cambisol	0.01–0.1	48–65	54	110–125	[39]
^13^C Glucose	Typic Hapludands	0.005	20–35	14	10–22	[75]
^14^C Glucose	Luvic Chernozem	0.005–0.5	25–50	12	(−87)–60	[31]
^14^C Cellulose	Gleyic Cambisol	0.04	29	103	25	[76]
^13^C Cellulose	Sandy silt	0.05	64–73	70	21–32	[77]
^14^C Cellulose	Sandy loam	0.5−1.2	52–75	90	28–37	[78]
^14^C Straw (wheat)	Sandy loam	0.5–1.2	24–33	32	7–9	[78]
^14^C Straw (rye)	Typic Kanadult	0.04	21–23	49	11	[79]
^13^C Straw (maize)	Fluvisol	0.2–0.3	16–18	250	9.1	[80]
^13^C Straw (rice)	Ferralic Cambisol	0.5	34–45	70	43–122	[81]
^13^C PBSA (bioplastic)	Haplic Chernozem	5	4.1–5.4	80	100–132	Present study

## Data Availability

Not applicable.

## Supplementary Materials

The following supporting information can be downloaded at: https://www.mdpi.com/article/10.3390/polym15030660/s1, Table S1. The statistic parameters of analysis of variance (ANOVA) of the CO_2_ emission of treatments during the experiment (correspond to Figure 2); Table S2. The statistic parameters of analysis of variance (ANOVA) of the contribution of three fractions in the decomposition rate (plastic, soil and priming) during the incubation experiment (correspond to Figure 3a); Table S3. The statistic parameters of analysis of variance (ANOVA) of the proportion of three fractions (plastic, soil and priming) during the incubation experiment (correspond to the Figure 4b).

## Abbreviations

C: N ratio: Carbon:Nitrogen ratio; PBAT: Poly(butylene adipate terephthalate); PBS: Poly(butylene succinate); PBSA: Poly(butylene succinate-co-adipate); BBP: Bio-based and biodegradable plastic; PE: priming effect; SOM: soil organic matter.

## References

[1] Yaeko S., Fumikazu A., Rumiko N., Takashi K. (2010). A novel method to discriminate between plant- and petroleum-derived plastics by stable carbon isotope analysis. Chem. Lett..

[2] Zumstein M.T., Narayan R., Kohler H.-P.E., McNeill K., Sander M. (2019). Dos and do nots when assessing the biodegradation of plastics. Environ. Sci. Technol..

[3] Liwarska-Bizukojc E. (2021). Effect of (bio)plastics on soil environment: A review. Sci. Total Environ..

[4] Volova T.G., Gladyshev M.I., Trusova M.Y., Zhila N.O., Kartushinskaya M.V. (2004). Degradation of bioplastics in natural environment. Dokl. Biol. Sci..

[5] Van den Oever M., Molenveld K., Zee M., Bos H. (2017). Bio-Based and Biodegradable Plastics—Facts and Figures. Focus on Food Packaging in the Netherlands.

[6] Iwata T. (2015). Biodegradable and bio-based polymers: Future prospects of eco-friendly plastics. Angew. Chem. Int. Ed..

[7] Haider T.P., Völker C., Kramm J., Landfester K., Wurm F.R. (2019). Plastics of the future? The impact of biodegradable polymers on the environment and on society. Angew. Chem. Int. Ed..

[8] Tanunchai B., Kalkhof S., Guliyev V., Wahdan S.F.M., Krstic D., Schädler M., Geissler A., Glaser B., Buscot F., Blagodatskaya E. (2022). Nitrogen fixing bacteria facilitate microbial biodegradation of a bio-based and biodegradable plastic in soils under ambient and future climatic conditions. Environ. Sci. Process. Impacts.

[9] Shinozaki Y., Morita T., Cao X.-h., Yoshida S., Koitabashi M., Watanabe T., Suzuki K., Sameshima-Yamashita Y., Nakajima-Kambe T., Fujii T. (2013). Biodegradable plastic-degrading enzyme from Pseudozyma antarctica: Cloning, sequencing, and characterization. Appl. Microbiol. Biotechnol..

[10] Fu Y., Wu G., Bian X., Zeng J., Weng Y. (2020). Biodegradation behavior of poly(butylene adipate-co-terephthalate) (PBAT), poly(lactic acid) (PLA), and their blend in freshwater with sediment. Molecules.

[11] Purahong W., Wahdan S.F.M., Heinz D., Jariyavidyanont K., Sungkapreecha C., Tanunchai B., Sansupa C., Sadubsarn D., Alaneed R., Heintz-Buschart A. (2021). Back to the future: Decomposability of a biobased and biodegradable plastic in field soil environments and its microbiome under ambient and future climates. Environ. Sci. Technol..

[12] Zumstein M.T., Schintlmeister A., Nelson T.F., Baumgartner R., Woebken D., Wagner M., Kohler H.-P.E., McNeill K., Sander M. (2018). Biodegradation of synthetic polymers in soils: Tracking carbon into CO_2_ and microbial biomass. Sci. Adv..

[13] Steinmetz Z., Wollmann C., Schaefer M., Buchmann C., David J., Tröger J., Muñoz K., Frör O., Schaumann G.E. (2016). Plastic mulching in agriculture. Trading short-term agronomic benefits for long-term soil degradation?. Sci. Total Environ..

[14] Castillo-Díaz F.J., Belmonte-Ureña L.J., Camacho-Ferre F., Tello-Marquina J.C. (2021). The management of agriculture plastic waste in the framework of circular economy. Case of the Almeria Greenhouse (Spain). Int J Environ. Res Public Health.

[15] Ramos L., Berenstein G., Hughes E.A., Zalts A., Montserrat J.M. (2015). Polyethylene film incorporation into the horticultural soil of small periurban production units in Argentina. Sci. Total Environ..

[16] Bandopadhyay S., Martin-Closas L., Pelacho A.M., DeBruyn J.M. (2018). Biodegradable plastic mulch films: Impacts on soil microbial communities and ecosystem functions. Front. Microbiol..

[17] Venkatesh S., Mahboob S., Govindarajan M., Al-Ghanim K.A., Ahmed Z., Al-Mulhm N., Gayathri R., Vijayalakshmi S. (2021). Microbial degradation of plastics: Sustainable approach to tackling environmental threats facing big cities of the future. J. King Saud Univ.—Sci..

[18] Abrusci C., Pablos J.L., Corrales T., López-Marín J., Marín I., Catalina F. (2011). Biodegradation of photo-degraded mulching films based on polyethylenes and stearates of calcium and iron as pro-oxidant additives. Int. Biodeterior. Biodegrad..

[19] Lozano Y.M., Rillig M.C. (2020). Effects of microplastic fibers and drought on plant communities. Environ. Sci. Technol..

[20] Pathan S.I., Arfaioli P., Bardelli T., Ceccherini M.T., Nannipieri P., Pietramellara G. (2020). Soil Pollution from micro- and nanoplastic debris: A hidden and unknown biohazard. Sustainability.

[21] MacLeod M., Arp H.P.H., Tekman M.B., Jahnke A. (2021). The global threat from plastic pollution. Science.

[22] Hoshino A., Sawada H., Yokota M., Tsuji M., Fukuda K., Kimura M. (2001). Influence of weather conditions and soil properties on degradation of biodegradable plastics in soil. Soil Sci. Plant Nutr..

[23] Moog D., Schmitt J., Senger J., Zarzycki J., Rexer K.-H., Linne U., Erb T., Maier U.G. (2019). Using a marine microalga as a chassis for polyethylene terephthalate (PET) degradation. Microb. Cell Factories.

[24] Bhuyar P., Sundararaju S., Feng H.X., Rahim M.H.A., Muniyasamy S., Maniam G.P., Govindan N. (2021). Evaluation of microalgae’s plastic biodeterioration property by a consortium of *Chlorella* sp. and *Cyanobacteria* sp. Environ. Res. Eng. Manag..

[25] Thielen M. (2019). Bioplastics: Plants and Crops Raw Materials Products.

[26] Abdul-Latif N.-I.S., Ong M.Y., Nomanbhay S., Salman B., Show P.L. (2020). Estimation of carbon dioxide (CO_2_) reduction by utilization of algal biomass bioplastic in Malaysia using carbon emission pinch analysis (CEPA). Bioengineered.

[27] Folino A., Karageorgiou A., Calabrò P., Komilis D. (2020). Biodegradation of wasted bioplastics in natural and industrial environments: A Review. Sustainability.

[28] Emadian S.M., Onay T.T., Demirel B. (2017). Biodegradation of bioplastics in natural environments. Waste Manag..

[29] Blagodatskaya E., Kuzyakov Y. (2013). Active microorganisms in soil: Critical review of estimation criteria and approaches. Soil Biol. Biochem..

[30] Kuzyakov Y., Friedel J., Stahr K. (2000). Review of mechanisms and quantification of priming effects. Soil Biol. Biochem..

[31] Blagodatskaya E.V., Blagodatsky S.A., Anderson T.H., Kuzyakov Y. (2007). Priming effects in Chernozem induced by glucose and N in relation to microbial growth strategies. Appl. Soil Ecol..

[32] Blagodatskaya E., Kuzyakov Y. (2008). Mechanisms of real and apparent priming effects and their dependence on soil microbial biomass and community structure: Critical review. Biol. Fertil. Soils.

[33] Chen R., Senbayram M., Blagodatsky S., Myachina O., Dittert K., Lin X., Blagodatskaya E., Kuzyakov Y. (2014). Soil C and N availability determine the priming effect: Microbial N mining and stoichiometric decomposition theories. Glob. Chang. Biol..

[34] Bei S., Li X., Kuyper T.W., Chadwick D.R., Zhang J. (2022). Nitrogen availability mediates the priming effect of soil organic matter by preferentially altering the straw carbon-assimilating microbial community. Sci. Total Environ..

[35] Rillig M.C., Leifheit E., Lehmann J. (2021). Microplastic effects on carbon cycling processes in soils. PLOS Biol..

[36] Zhang S., Wang J., Hao X. (2020). Fertilization accelerates the decomposition of microplastics in mollisols. Sci. Total Environ..

[37] Berto D., Rampazzo F., Gion C., Noventa S., Ronchi F., Traldi U., Giorgi G., Cicero A.M., Giovanardi O. (2017). Preliminary study to characterize plastic polymers using elemental analyser/isotope ratio mass spectrometry (EA/IRMS). Chemosphere.

[38] Amelung W., Brodowski S., Sandhage-Hofmann A., Bol R. (2008). Chapter 6: Combining biomarker with stable isotope analyses for assessing the transformation and turnover of soil organic matter. Advances in Agronomy.

[39] Blagodatskaya E., Yuyukina T., Blagodatsky S., Kuzyakov Y. (2011). Three-source-partitioning of microbial biomass and of CO_2_ efflux from soil to evaluate mechanisms of priming effects. Soil Biol. Biochem..

[40] Blagodatskaya E., Yuyukina T., Blagodatsky S., Kuzyakov Y. (2011). Turnover of soil organic matter and of microbial biomass under C3–C4 vegetation change: Consideration of ^13^C fractionation and preferential substrate utilization. Soil Biol. Biochem..

[41] Glaser B., Gross S. (2005). Compound-specific δ^13^C analysis of individual amino sugars—A tool to quantify timing and amount of soil microbial residue stabilization. Rapid Commun. Mass Spectrom..

[42] Glaser B. (2005). Compound-specific stable-isotope (δ^13^C) analysis in soil science. J. Plant Nutr. Soil Sci..

[43] Boschker H., Middelburg J. (2002). Boschker HTS, Middelburg JJ.. Stable isotopes and biomarkers in microbial ecology. FEMS Microbiol. Ecol..

[44] Hobbie A.E., Werner R.A. (2004). Intramolecular, compound-specific, and bulk carbon isotope patterns in C3 and C4 plants: A review and synthesis. New Phytol..

[45] ŠantRůČková H., Bird M.I., Lloyd J. (2000). Microbial processes and carbon-isotope fractionation in tropical and temperate grassland soils. Funct. Ecol..

[46] Formánek P., Ambus P. (2004). Assessing the use of δ^13^C natural abundance in separation of root and microbial respiration in a Danish beech (*Fagus sylvatica* L.) forest. Rapid Commun. Mass Spectrom. RCM.

[47] Boström B., Comstedt D., Ekblad A. (2007). Isotope fractionation and ^13^C enrichment in soil profiles during the decomposition of soil organic matter. Oecologia.

[48] Breecker D., Bergel S., Nadel M., Tremblay M., Osuna Orozco R., Larson T., Sharp Z. (2014). Minor stable carbon isotope fractionation between respired carbon dioxide and bulk soil organic matter during laboratory incubation of topsoil. Biogeochemistry.

[49] Werth M., Kuzyakov Y. (2010). ^13^C fractionation at the root–microorganisms–soil interface: A review and outlook for partitioning studies. Soil Biol. Biochem..

[50] Volk M., Bassin S., Lehmann M.F., Johnson M.G., Andersen C.P. (2018). ^13^C isotopic signature and C concentration of soil density fractions illustrate reduced C allocation to subalpine grassland soil under high atmospheric N deposition. Soil Biol. Biochem..

[51] Altermann M., Rinklebe J., Merbach I., Körschens M., Langer U., Hofmann B. (2005). Chernozem—Soil of the Year 2005. J. Plant Nutr. Soil Sci..

[52] Schädler M., Buscot F., Klotz S., Reitz T., Durka W., Bumberger J., Merbach I., Michalski S.G., Kirsch K., Remmler P. (2019). Investigating the consequences of climate change under different land-use regimes: A novel experimental infrastructure. Ecosphere.

[53] Tanunchai B., Juncheed K., Wahdan S.F.M., Guliyev V., Udovenko M., Lehnert A.-S., Alves E.G., Glaser B., Noll M., Buscot F. (2021). Analysis of microbial populations in plastic–soil systems after exposure to high poly(butylene succinate-co-adipate) load using high-resolution molecular technique. Environ. Sci. Eur..

[54] Guliyev V., Tanunchai B., Noll M., Buscot F., Purahong W., Blagodatskaya E. (2022). Links among microbial communities, soil properties and functions: Are fungi the sole players in decomposition of bio-based and biodegradable plastic?. Polymers.

[55] Nordgren A. (1988). Apparatus for the continuous, long-term monitoring of soil respiration rate in large numbers of samples. Soil Biol. Biochem..

[56] Farquhar G.D., Ehleringer J.R., Hubick K.T. (1989). Carbon isotope discrimination and photosynthesis. Annu. Rev. Plant Biol..

[57] Balesdent J., Mariotti A. (1996). Measurement of Soil Organic Matter Turnover Using ^13^C Natural Abundance.

[58] Potthoff M., Loftfield N., Buegger F., Wick B., John B., Joergensen R.G., Flessa H. (2003). The determination of δ^13^C in soil microbial biomass using fumigation-extraction. Soil Biol. Biochem..

[59] Tu K., Dawson T., Flanagan L.B., Ehleringer J.R., Pataki D.E. (2005). Partitioning ecosystem respiration using stable carbon isotope analyses of CO_2_. Stable Isotopes and Biosphere Atmosphere Interactions.

[60] Pelz O., Abraham W.-R., Saurer M., Siegwolf R., Zeyer J. (2005). Microbial assimilation of plant-derived carbon in soil traced by isotope analysis. Biol. Fertil. Soils.

[61] Ghashghaie J., Duranceau M., Badeck F.-W., Cornic G., Adeline M.-T., Deleens E. (2001). δ^13^C of CO_2_ respired in the dark in relation to δ^13^C of leaf metabolites: Comparison between Nicotiana sylvestris and Helianthus annuus under drought. Plant Cell Environ..

[62] Werth M., Kuzyakov Y. (2009). Three-source partitioning of CO_2_ efflux from maize field soil by ^13^C natural abundance. J. Plant Nutr. Soil Sci..

[63] Ågren G.I., Bosatta E., Balesdent J. (1996). Isotope discrimination during decomposition of organic matter: A theoretical analysis. Soil Sci. Soc. Am. J..

[64] Crow S.E., Sulzman E.W., Rugh W.D., Bowden R.D., Lajtha K. (2006). Isotopic analysis of respired CO_2_ during decomposition of separated soil organic matter pools. Soil Biol. Biochem..

[65] Salomé C., Nunan N., Pouteau V., Lerch T.Z., Chenu C. (2010). Carbon dynamics in topsoil and in subsoil may be controlled by different regulatory mechanisms. Glob. Chang. Biol..

[66] Qiao N., Xu X., Hu Y., Blagodatskaya E., Liu Y., Schaefer D., Kuzyakov Y. (2016). Carbon and nitrogen additions induce distinct priming effects along an organic-matter decay continuum. Sci. Rep..

[67] Moran K.K., Six J., Horwath W.R., van Kessel C. (2005). Role of mineral-nitrogen in residue decomposition and stable soil organic matter formation. Soil Sci. Soc. Am. J..

[68] Gómez E.F., Michel F.C. (2013). Biodegradability of conventional and bio-based plastics and natural fiber composites during composting, anaerobic digestion and long-term soil incubation. Polym. Degrad. Stab..

[69] Blagodatsky S., Larionova A., Yevdokimov I. (1993). Effect of mineral nitrogen on the respiration rate and growth efficiency of soil microorganisms. Eurasian Soil Sci..

[70] Shahbaz M., Kuzyakov Y., Sanaullah M., Heitkamp F., Zelenev V., Kumar A., Blagodatskaya E. (2017). Microbial decomposition of soil organic matter is mediated by quality and quantity of crop residues: Mechanisms and thresholds. Biol. Fertil. Soils.

[71] Nottingham A.T., Griffiths H., Chamberlain P.M., Stott A.W., Tanner E.V.J. (2009). Soil priming by sugar and leaf-litter substrates: A link to microbial groups. Appl. Soil Ecol..

[72] Liming Y., Zhang T., Dijkstra F., Huo C., Wang P., Cheng W. (2021). Priming effect varies with root order: A case of Cunninghamia Lanceolata. Soil Biol. Biochem..

[73] Wang Q., He T., Liu J. (2016). Litter input decreased the response of soil organic matter decomposition to warming in two subtropical forest soils. Sci. Rep..

[74] Chao L., Liu Y., Freschet G.T., Zhang W., Yu X., Zheng W., Guan X., Yang Q., Chen L., Dijkstra F.A. (2019). Litter carbon and nutrient chemistry control the magnitude of soil priming effect. Funct. Ecol..

[75] Brant J.B., Sulzman E.W., Myrold D.D. (2006). Microbial community utilization of added carbon substrates in response to long-term carbon input manipulation. Soil Biol. Biochem..

[76] Blagodatskaya E., Khomyakov N., Myachina O., Bogomolova I., Blagodatsky S., Kuzyakov Y. (2014). Microbial interactions affect sources of priming induced by cellulose. Soil Biol. Biochem..

[77] Fontaine S., Bardoux G., Abbadie L., Mariotti A. (2004). Carbon input to soil may decrease soil carbon content. Ecol. Lett..

[78] Dalenberg J.W., Jager G. (1989). Priming effect of some organic additions to ^14^C-labelled soil. Soil Biol. Biochem..

[79] Cheng W., Coleman D.C. (1990). Effect of living roots on soil organic matter decomposition. Soil Biol. Biochem..

[80] Qiu Q., Wu L., Ouyang Z., Li B., Xu Y., Wu S., Gregorich E.G. (2016). Priming effect of maize residue and urea N on soil organic matter changes with time. Appl. Soil Ecol..

[81] Wu L., Zhang W., Wei W., He Z., Kuzyakov Y., Bol R., Hu R. (2019). Soil organic matter priming and carbon balance after straw addition is regulated by long-term fertilization. Soil Biol. Biochem..

