# Supplementary material for: Degradation of Bio-Based and Biodegradable Plastic and Its Contribution to Soil Organic Carbon Stock
Source: Polymers (Basel). 2023 Jan 28;15(3):660. doi: 10.3390/polym15030660 (PMC9919936; doi:10.3390/polym15030660)
Supplement: Supplementary file 1 [file polymers-15-00660-s001.zip › polymers-2114251 supplementary material update.pdf]

# Supplementary material

## Degradation of bio-based and biodegradable plastics and its contribution to soil organic carbon stock

**Vusal Guliyev [1,2,3], Benjawan Tanunchai [1,4], Maria Udovenko [1], Oleg Menyailo [5], Bruno Glaser [6,7], Witoon Purahong [1,*], François Buscot [1,7] and Evgenia Blagodatskaya [1,*]**

[1] UFZ-Helmholtz Centre for Environmental Research, Department of Soil Ecology, 06120 Halle (Saale), Germany; vusal.guliyev@ufz.de (V.G.); tanunchai.benjawan@ufz.de (B.T.); udovenkomasha94@mail.ru (M.U.); francois.buscot@ufz.de (F.B.)
[2] Department of Biology, University of Leipzig, 04103 Leipzig, Germany
[3] Institute of Soil Science and Agro Chemistry, ANAS, AZ1073 Baku, Azerbaijan
[4] Bayreuth Center of Ecology and Environmental Research (BayCEER), University of Bayreuth, 95448 Bayreuth, Germany
[5] Joint FAO/IAEA Centre of Nuclear Techniques in Food and Agriculture, Soil and Water Management and Crop Nutrition Laboratory, 2444 Seibersdorf, Austria; o.meniailo@iaea.org
[6] Department of Soil Biogeochemistry, Martin Luther University Halle-Wittenberg, 06120 Halle (Saale), Germany; bruno.glaser@landw.uni-halle.de
[7] German Centre for Integrative Biodiversity Research (iDiv), Halle-Jena-Leipzig, 04103 Leipzig, Germany
***** Correspondence: witoon.purahong@ufz.de, witoon.purahong@gmail.com (W.P.); evgenia.blagodatskaya@ufz.de (E.B.)

**Table S1.** The statistic parameters of analysis of variance (ANOVA) of the $CO_2$ emission of treatments during the experiment (corresponds to the Fig. 2). Asterisks indicate significant differences (with * for p < 0.05, ** for p < 0.01 and *** for p < 0.001).

| **Source** | **Sum of squares** | **df** | ***F*-value** | ***p*-value** |
|---|---|---|---|---|
| Time | 1011400976 | 13 | 171.62 | < 0.001*** |
| Treatment | 70405394 | 3 | 516.36 | < 0.001*** |
| Time: Treatment | 39606137 | 39 | 22.34 | < 0.001*** |

**Table S2.** The statistic parameters of analysis of variance (ANOVA) of the contribution of three fractions in the decomposition rate (plastic, soil, and priming) during the incubation experiment (corresponds to the Fig. 3a). Asterisks indicate significant differences (with * for p < 0.05, ** for p < 0.01 and *** for p < 0.001).

| Fraction | Treatment | Time | Treatment * Time |
|---|---|---|---|
| soil | F= 20.30,p<0.001*** | F= 22.26,p<0.001*** | F=0.82,p>0.05 |
| priming | F=3.66, p>0.05 | F=5.59, p<0.001*** | F=4.89, p<0.01** |
| plastic | F=9.08, p<0.01** | F=2.43, p<0.05* | F=15.62, p>0.001*** |

**Table S3.** The statistic parameters of analysis of variance (ANOVA) of the proportion of three fractions (plastic, soil, and priming) during the incubation experiment (corresponds to the Fig. 4b). Asterisks indicate significant differences (with * for p < 0.05, ** for p < 0.01 and *** for p < 0.001).

| Fraction | Treatment | Time | Treatment * Time |
|---|---|---|---|
| soil | F=0.88, p>0,05 | F=1.78, p>0,05 | F= 4.76,p<0,01 ** |
| priming | F=2.64, p>0.05 | F=3.47, p<0,01 ** | F= 5.41,p<0,01 ** |
| plastic | F=5,79,p<0.05 * | F=4,36,p<0.01 ** | F=8,38,p<0,001 *** |
